# Auricular Tophaceous Gout Resembling Calcinosis Cutis in a Young Male: A Case Report

**DOI:** 10.7759/cureus.95102

**Published:** 2025-10-21

**Authors:** Adnan Ahmad, Alsadat Mosbeh, Abrar Abbas, Nasser Alrumaidhi, Abeer Albazali

**Affiliations:** 1 Dermatology, Farwaniya Hospital, Farwaniya, KWT; 2 Dermatology, Kuwait Institute for Medical Specializations, Sulibekhat, KWT; 3 Dermatology/Dermatopathology, Faculty of Medicine, Al-Azhar University, Cairo, EGY; 4 Internal Medicine, Farwaniya Hospital, Farwaniya, KWT

**Keywords:** calcinosis cutis, dermatology, gout, hyperuricemia, rheumatology, tophi

## Abstract

Gout is an inflammatory disorder resulting from the deposition of monosodium urate (MSU) crystals in body tissues. Chronic or undertreated gout can progress to tophaceous gout. Although it most commonly affects the joints, auricular involvement is rare and can be misdiagnosed as other conditions, such as calcinosis cutis. We report a case of a 36-year-old male with a two-year history of slowly growing, painless, hard, white nodules on both auricles. Laboratory investigations revealed hyperuricemia, and histopathological examination demonstrated features consistent with gout tophi. Based on the clinical, biochemical, and histopathological findings, a final diagnosis of auricular tophaceous gout was established. This case highlights the diagnostic challenges posed by atypical presentations of gout, particularly when mimicking calcinosis cutis. Auricular tophi should be considered in patients with persistent hard auricular nodules and hyperuricemia. Clinical, biochemical, and histopathological correlation is essential for establishing a definitive diagnosis.

## Introduction

Gout is one of the most common inflammatory disorders, caused by the deposition of monosodium urate (MSU) crystals in body tissues [1]. In long-standing or under-treated gout, MSU crystal deposits can progress into the formation of palpable tophi, typically containing a white chalky material, around joints, soft tissues, and skin [2]. While tophaceous gout most commonly forms in periarticular regions, atypical locations such as the auricular appendages are rare and often underrecognized [2,3]. The differentiation between gout tophi and calcinosis cutis can be challenging due to the overlapping clinical features, including hard, slow-growing, and whitish nodules [2,4,5]. Herein, we report a diagnostically challenging case of auricular tophaceous gout mimicking calcinosis cutis in a young male with psoriatic arthritis, emphasizing the importance of clinical, biochemical, and histopathological correlation in the diagnosis of such atypical presentations.

## Case presentation

**Figure 1:**
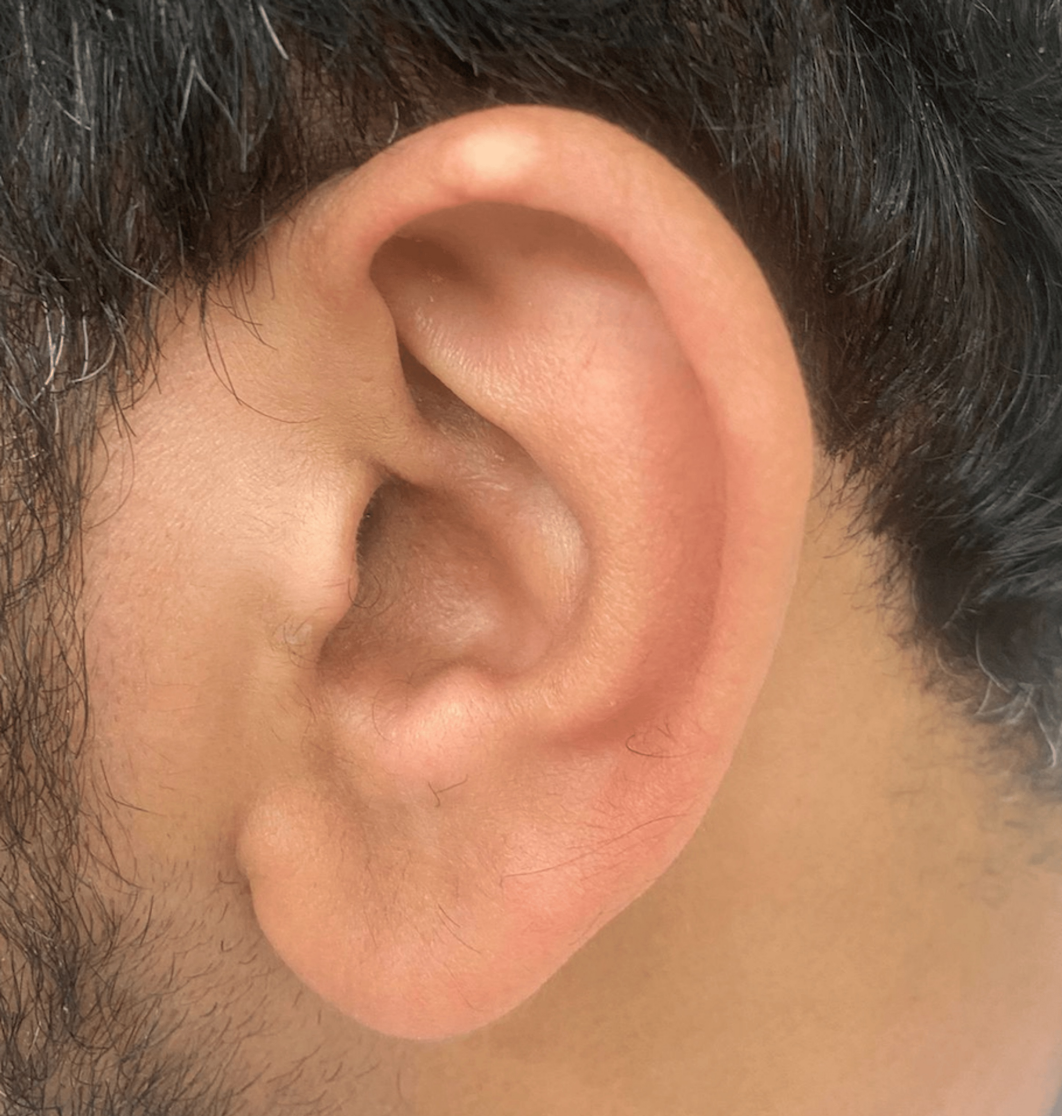
A well-defined pearly white nodule on the helix of the left ear.

A 36-year-old male was referred from the rheumatology department for evaluation of bilateral auricular nodules, with an initial suspicion of calcinosis cutis by the rheumatologists. The nodules had been present for approximately two years and were slowly progressive in size. They were described as painless, non-pruritic, and non-bleeding. The patient’s past medical history was significant for psoriatic arthritis (PsA), diagnosed in 2021, for which he is being treated with guselkumab. He reported recurrent episodes of joint pain involving the ankles and knees over the past four years. There was no history of trauma, recent infections, ear pain, or tinnitus. He denied systemic symptoms such as fever, weight loss, abdominal pain, urinary symptoms, or changes in bowel habits. Past surgical history, family history, and social history were unremarkable. Physical examination revealed a single, well-defined, pearly-white nodule on the helix of the left ear (Figure 1).

**Figure 2:**
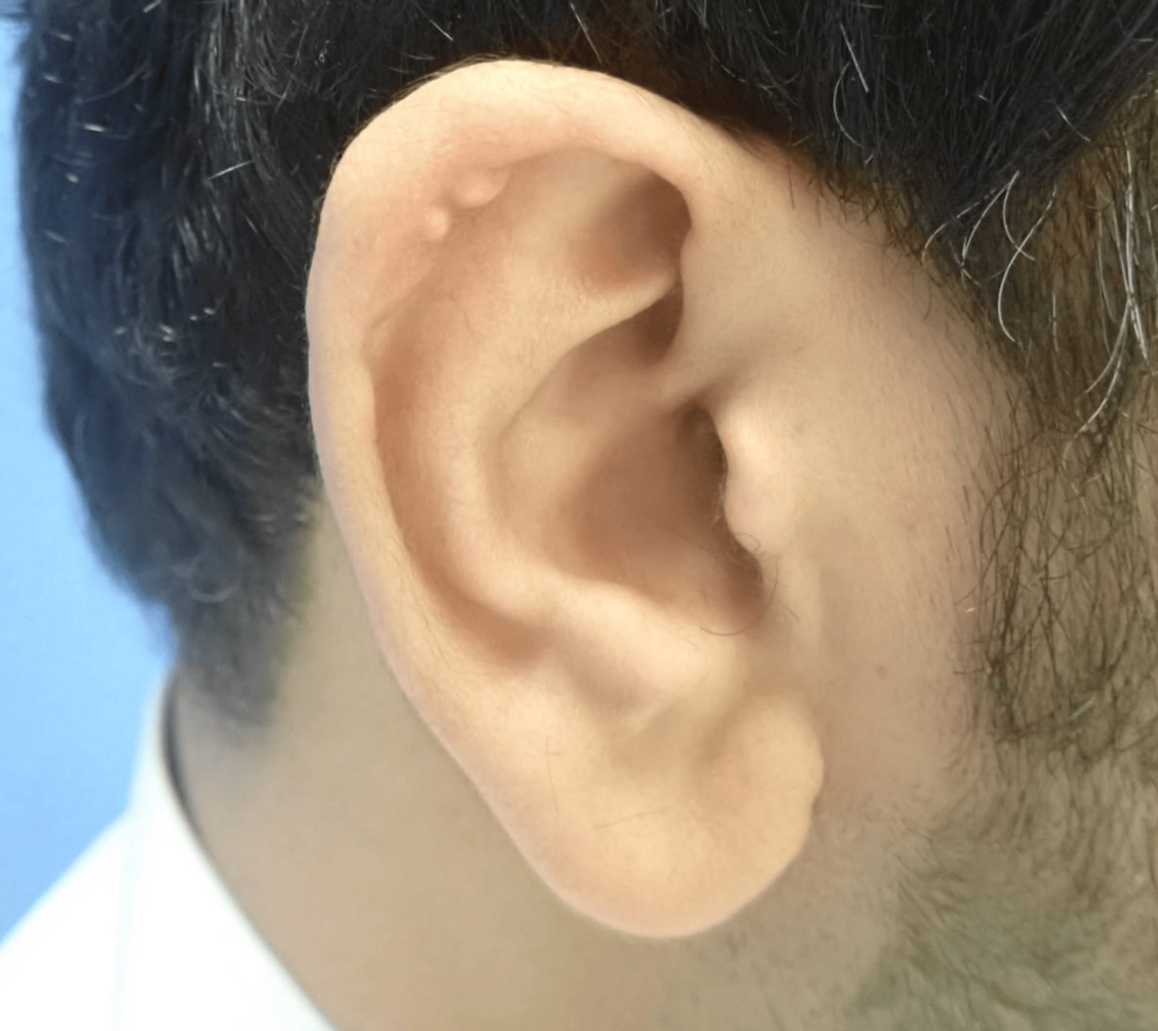
Two well-defined, pearly white nodules on the helix of the right ear.

On the right ear, two similar nodules were identified on the helix (Figure 2). All lesions were non-tender and hard on palpation, with no similar lesions observed elsewhere on the body. Our differential diagnosis included calcinosis cutis, xanthoma, gout tophi, keloid scars, and chondrodermatitis nodularis helicis.

Laboratory investigations are summarized in Table 1. Notable abnormalities included elevated inflammatory markers (ESR and CRP) and hyperuricemia, with normal corrected calcium and insufficient calcidiol levels. Additional investigations including complete blood count, liver function tests, lipid profile, thyroid function tests, hemoglobin A1C, and parathyroid hormone-were all within normal limits.

**Table 1:** Patient's laboratory test results

Test	Patient result	Reference range
Erythrocyte sedimentation rate (ESR)	57 mm/hr	< 15 mm/hr
C-reactive protein (CRP)	128 mg/L	< 10 mg/L
Uric acid	683 µmol/L	208 – 428 µmol/L
Corrected calcium	2.41 mmol/L	2.2 – 2.6 mmol/L
Calcidiol (25-hydroxyvitamin D)	53 nmol/L	75 – 125 nmol/L

**Figure 3:**
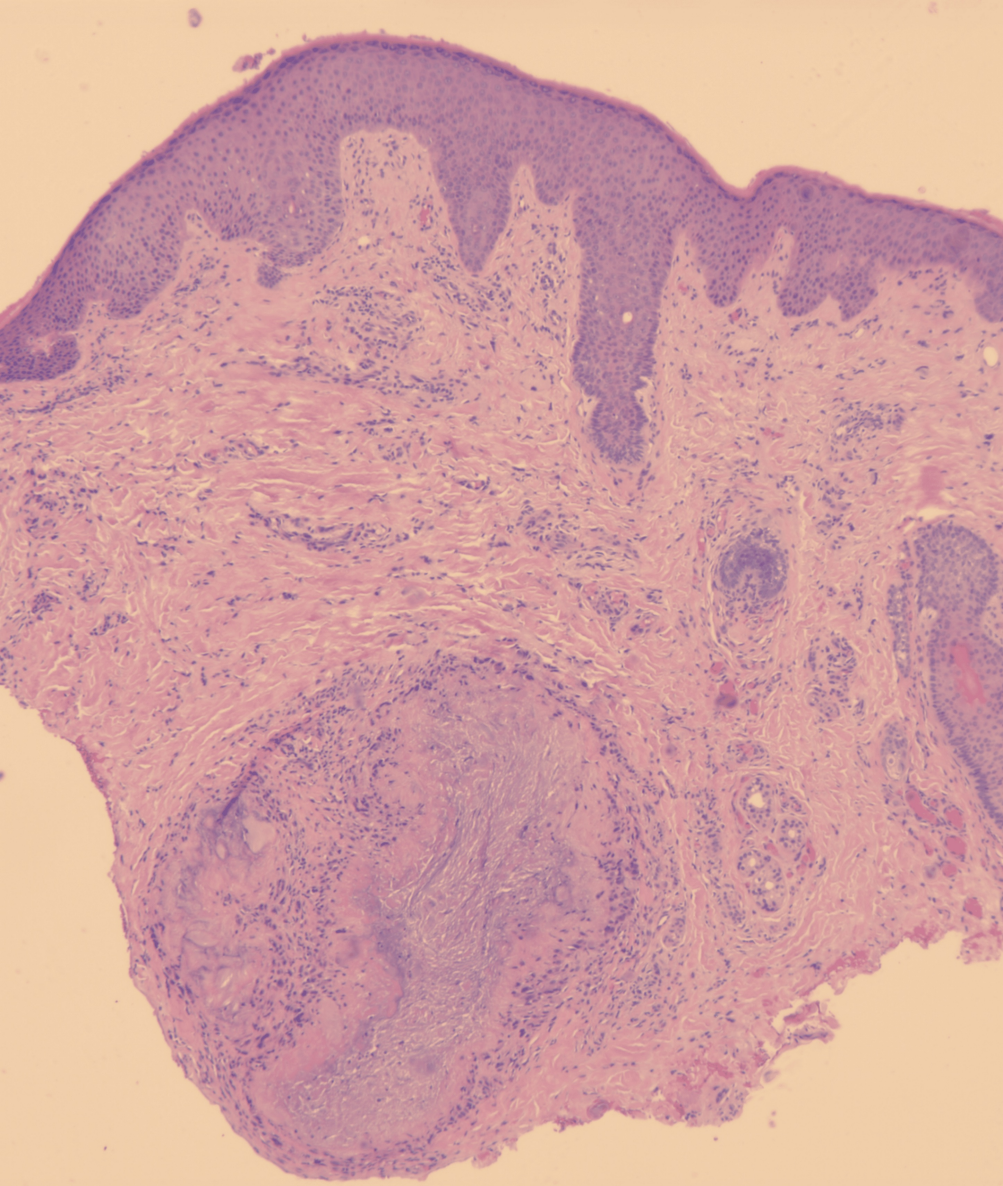
Histopathology of the skin biopsy showing hyperkeratosis and acanthosis of the epidermis with pale-pink deposits in the dermis, (H&E, x2.5) H&E: hematoxylin and eosin

A punch biopsy was subsequently obtained from the left ear nodule. Histopathological examination with hematoxylin and eosin (H&E) staining demonstrated epidermal hyperkeratosis and acanthosis with elongation of the rete ridges (Figure 3). The deep dermis contained deposits of amorphous, pale-pink material surrounded by a rim of granulomatous inflammatory infiltrates (Figure 3).

**Figure 4:**
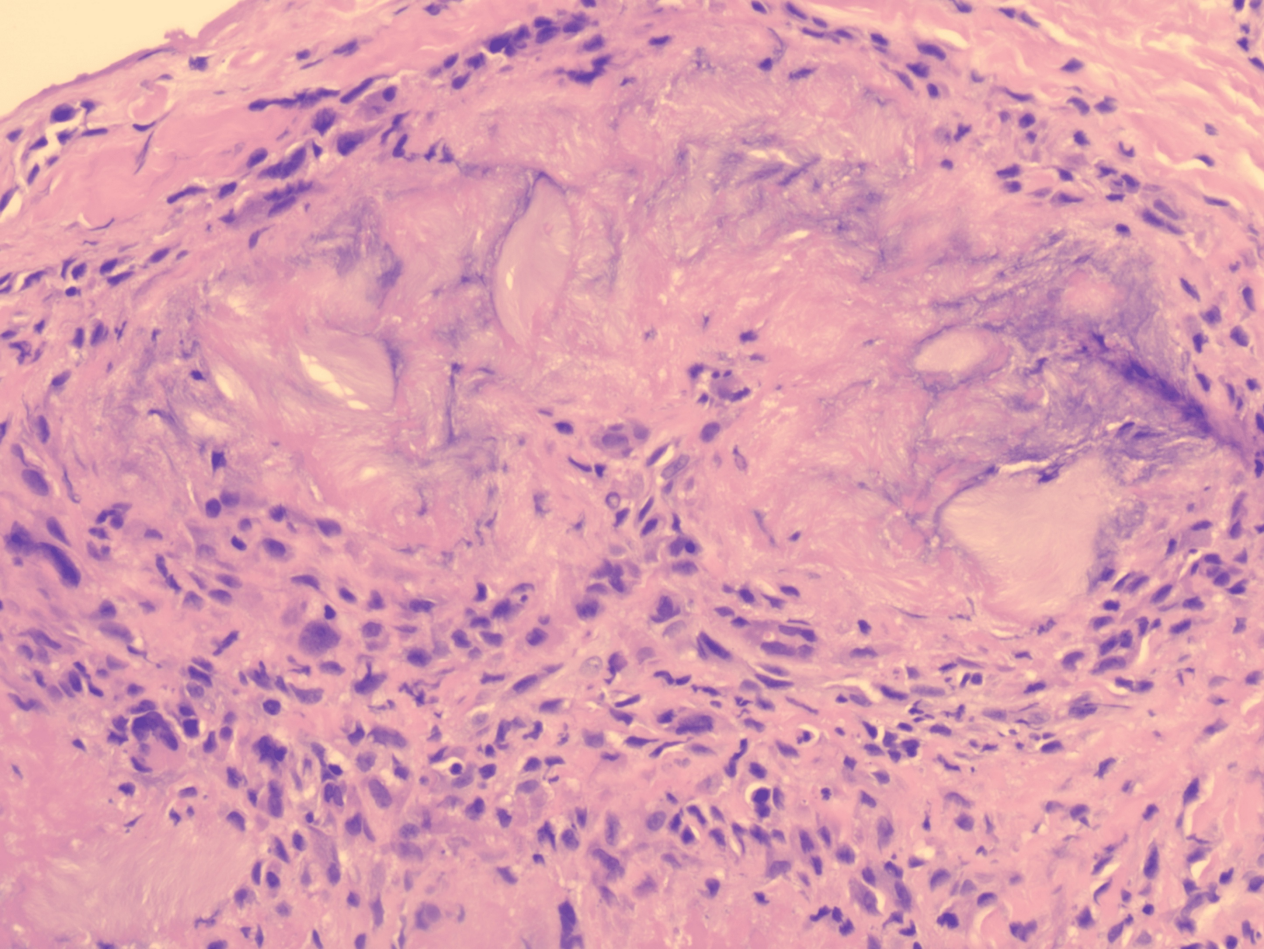
Rim of granulomatous infiltrate surrounding the needle-shaped clefts of the dissolved urate crystals, (H&E, x200) H&E: hematoxylin and eosin

High-power magnification revealed feathery, needle-shaped clefts within the deposits, characteristic of dissolved urate crystals due to formalin fixation (Figure 4). Therefore, a final diagnosis of auricular tophaceous gout was established based on clinico-pathological correlation in addition to the hyperuricemia. The final diagnosis was discussed with the treating rheumatologist, and the patient was started on oral allopurinol 100 mg once daily with a plan to increase the dose gradually by the treating rheumatologist.

## Discussion

This case highlights the diagnostic complexity that can arise when gout tophi present in atypical locations such as the auricles. Tophaceous gout is classically associated with chronic hyperuricemia or under-treated gout and most frequently involves the metatarsophalangeal, ankle, knee, and arm joints [6]. Auricular gout tophi, while reported in the literature, remain rare and often overlooked due to their resemblance to other conditions such as calcinosis cutis, rheumatoid nodules, and xanthoma [3]. Clinically, both gout tophi and calcinosis cutis can present as firm or hard, whitish nodules with a chronic indolent course [2,5]. In calcinosis cutis, the nodules result from the deposition of insoluble calcium salts in the skin and subcutaneous tissue, and it is commonly associated with connective tissue diseases such as dermatomyositis, lupus erythematosus, and systemic sclerosis [7]. In this case, laboratory investigations showed normal serum levels of calcium, phosphate, and parathyroid hormone, with low vitamin D3 levels (calcidiol). The combination of these findings, along with the absence of recent trauma, systemic infection, or a co-existing connective tissue disease, made the diagnosis of calcinosis cutis less likely [5]. Comparatively, gout tophi result from the deposition of MSU crystals in the tissues and are mainly associated with hyperuricemia [1]. In this case, laboratory tests have revealed a significantly elevated level of serum uric acid (683 µmol/L), raising the suspicion of gout tophi. Histopathological evaluation of the skin biopsy revealed deposits of amorphous pale-pink material in the deep dermis surrounded by a rim of granulomatous inflammatory infiltrate. Within the deposits, needle-shaped clefts representing dissolved uric acid crystals due to formalin fixation were observed, which are characteristic histological features of gout tophi [8]. The classical calcium deposits associated with calcinosis cutis were not observed, excluding the diagnosis of calcinosis cutis. The highlighted clinical, biochemical, and histopathological features were all supportive of the diagnosis of tophaceous gout. Treatment should be started with urate-lowering medications, such as allopurinol, in addition to lifestyle adjustments to prevent joint deformities [9].

## Conclusions

Auricular tophaceous gout is a rare and often underrecognized manifestation of tophaceous gout, which may be mistaken for other conditions such as calcinosis cutis. In patients with underlying hyperuricemia, the development of progressive auricular nodules should prompt consideration of gout tophi. While clinical evaluation and laboratory testing provide important diagnostic clues, histopathological examination remains essential for confirming the diagnosis. Increased awareness of atypical presentations of tophaceous gout is needed to avoid misdiagnoses and improve patient care and outcomes. Proper treatment, coupled with a healthy lifestyle, must be started promptly since gout is a joint-deforming disease.
